# Evaluation of Non-Faradaic Impedimetric Parameters for IL-8 Detection Using Gold Interdigitated Electrode-Based Biosensors: Towards Early Detection of Newborn Disability

**DOI:** 10.3390/mi16040395

**Published:** 2025-03-28

**Authors:** Abdulelah S. Alrebaish, Layla O. Alnami, Joud M. Alshraim, Razan A. Alnghemshi, Alanoud A. Aljammaz, Amir Altinawi, Kholood K. Alhuthali, Hend Alfadul, Abdulaziz K. Assaifan

**Affiliations:** 1 Department of Biomedical Technology, College of Applied Medical Sciences, King Saud University, P.O. Box 10219, Riyadh 11433, Saudi Arabia; aalrebaish@ksu.edu.sa (A.S.A.); 442200544@student.ksu.edu.sa (L.O.A.); 442202177@student.ksu.edu.sa (J.M.A.); 443202756@student.ksu.edu.sa (R.A.A.); 443202645@student.ksu.edu.sa (A.A.A.); atinawi@ksu.edu.sa (A.A.); 444203379@student.ksu.edu.sa (K.K.A.); hend-7@live.com (H.A.); 2 King Salman Center for Disability Research, Riyadh 11614, Saudi Arabia; 3 Biological and Environmental Sensing Research Unit, King Abdullah Institute for Nanotechnology, King Saud University, P.O. Box 2455, Riyadh 11451, Saudi Arabia

**Keywords:** non-Faradaic biosensor, impedimetric parameters, interleukin-8, disability

## Abstract

Interleukin-8 (IL-8) is a critical biomarker associated with inflammation and disability in both adults and newborns. Conventional detection methods are often labor-intensive, time-consuming, and require highly trained personnel. Non-Faradaic impedimetric biosensors offer a label-free, rapid, and direct approach for IL-8 detection. While previous studies have primarily focused on capacitance and phase changes, the potential of other impedimetric parameters remains underexplored. In this study, a gold interdigitated electrode (Au-IDE)-based non-Faradaic biosensor was developed for IL-8 detection, evaluating multiple impedimetric parameters, including capacitance, impedance magnitude (Z_mod_), real impedance (Z_real_), and imaginary impedance (Z_imag_). Among these, Z_imag_ exhibited the lowest limit of detection (LoD) at 90 pg/mL, followed by Z_mod_ at 120 pg/mL, and capacitance at 140 pg/mL, all significantly below the clinical threshold of 600 pg/mL. In contrast, Z_real_ displayed the highest LoD at 1.3 ng/mL. Sensitivity analysis revealed that Z_imag_ provided the highest sensitivity at 13.1 kΩ/log (ng/mL), making it the most effective parameter for detecting IL-8 at low concentrations. The sensitivity of Z_mod_ and Z_real_ was lower, while capacitance sensitivity was measured at 20 nF/log (ng/mL). These findings highlight the importance of investigating alternative impedimetric parameters beyond capacitance to optimize biosensor performance for biomarker detection. This study demonstrates that non-Faradaic biosensors, despite their capacitive-based nature, can achieve enhanced sensitivity and detection limits by leveraging additional impedimetric parameters, offering a promising approach for rapid and effective IL-8 detection.

## 1. Introduction

Newborn disabilities refer to abnormalities that cause significant health and developmental challenges. They are strongly associated with increased mortality rates and long-term complications [1]. Genetic factors, prenatal exposures, and perinatal complications are all risk factors associated with developmental delays [2,3]. Early diagnosis and intervention can improve developmental outcomes for infants who are at high risk [4]. Different diagnostic and screening tools are available to detect disabilities to ensure better long-term health, cognitive development, and overall quality of life for affected children [5].

Traditional techniques rely on observing physical milestones such as proning, rolling, and posture maintenance through natural play and structured activities [6]. A widely used tool is the “Ages and Stages Questionnaire (ASQ)”, which evaluates motor, communication, problem solving, and social–emotional development in children. It is cost-effective, easy to apply, and effective for early detection. However, its limitations include potential false positives, reliance on parental input, and cultural variability [7]. Standardized assessment tools like the Bayley Scales of Infant and Toddler Development (BSID) provides insight for assessing development but may overlook cultural and linguistic differences. Together, these methods form a foundational framework for identifying developmental disabilities, despite their challenges [8].

Conditions associated with significant developmental disabilities, like neonatal encephalopathy, are linked to altered levels of pro-inflammatory markers such as interleukin-8 (IL-8) and granulocyte–macrophage colony-stimulating factor (GM-CSF) [8]. Research has shown that these altered biomarkers correlate to negative clinical outcomes [9]. IL-8 plays various roles in different physiological and pathological processes [10]. In auto-inflammatory disease, IL-8 amplifies the pro-inflammatory environment in various tissues [11]. In cancer, IL-8 stimulates tumor growth and metastasis invasion, acting as an autocrine growth factor [12]. IL-8 also shows a crucial role in wound healing by recruiting neutrophils to the injury site, initiating its activation and serving as a chemoattractant [13]. In addition, IL-8 promotes angiogenesis, an essential physiological process in wound healing that includes the progression of new blood vessels contributing to tissue repair and regeneration [14]. In the neonatal intensive care unit (NICU), IL-8 may serve as a critical marker for inflammation and infection [15]. As a pro-inflammatory cytokine, IL-8 is crucial in neonatal brain injury and long-term disabilities. Elevated IL-8 levels in neonates, particularly in preterm infants, are associated with an increased risk of cerebral palsy, cognitive delays, and behavioral disorders [16]. Monitoring this biomarker may help predict outcomes, allowing for early intervention and potentially improving long-term health and developmental outcomes [9]. A wide range of methods are used to monitor the blood levels of IL-8: IMMULITE 1000 Immunoassay System [17,18], BioPlex Multiplex Immunoassays [19], electrochemiluminescence immunoassay plates [20], and sandwich enzyme-linked immunosorbent assay (ELISA) kits [21]. Such methods are lengthy and timely and require specific instruments and lab experts, which beats the purpose of easily available, prompt assays [22]. To overcome these drawbacks, extensive research is ongoing to develop novel, rapid, and user-friendly techniques.

Biosensors have dramatically transformed the monitoring of disease and related biomarkers [23]. They have emerged as sophisticated analytical tools that have revolutionized disease diagnosis and health monitoring paradigms. The International Union of Pure and Applied Chemistry (IUPAC) defined biosensors as “A device that uses specific biochemical reactions mediated by isolated enzymes, immunosystems, tissues, organelles, or whole cells to detect chemical compounds usually by electrical, thermal, or optical signals” [24]. The evolution of biosensors has demonstrated remarkable progression, establishing these devices as integral components across multiple healthcare domains, including early disease detection, preventive medicine, rehabilitation protocols, and continuous patient monitoring systems [25]. Because of how straightforward and inexpensive they are, electrochemical non-Faradaic biosensors have been widely used for the direct analysis of a selection of different biomarkers associated with chronic diseases, infectious diseases, and cancer [26,27,28].

Non-Faradaic impedimetric biosensors mainly rely on alterations to double-layer capacitance at the biosensor/electrolyte interface. When antigens bind to antibodies immobilized onto surfaces, the surface area, surface roughness, or dielectric properties change, causing alterations to the double-layer capacitance. Research work on non-Faradaic biosensors mainly reported changes in capacitance, phase, and impedance magnitude [29,30]. Herein, we investigate thoroughly which parameters of gold interdigitated electrode (Au-IDE)-based non-Faradaic biosensors prove better sensitivity and limits of detection. Au-IDEs were immobilized with IL-8 antibodies via a standard biofunctionalization approach. Changes in capacitance, impedance magnitude (Z_mod_), real impedance (Z_real_), and imaginary impedance (Z_imag_) were observed when the functionalized biosensor was exposed to series concentrations of IL-8 antigens. The sensitivity and limit of detection (LoD) were obtained for each impedimetric parameter. The results reported in this work show that in-depth analysis is needed when using non-Faradaic impedimetric biosensors. Although the technique is based on capacitive changes at the electrode/electrolyte interface, other impedimetric parameters may show better performance than capacitance only. The reported work shows a detailed analysis of the non-Faradaic biosensor response to the IL-8 antigen. In addition, it provides an easy route for the direct mass screening of biomarkers associated with inflammation and disability.

## 2. Materials and Methods

### 2.1. Materials

**Figure 1:**
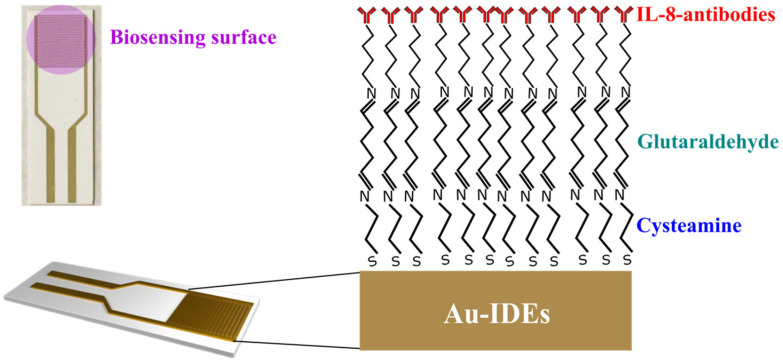
Photograph of Au-IDEs and biofunctionalization of the non-Faradaic biosensor.

Interdigitated gold electrodes on a plastic substrate (Metrohm Dropsense, Ref. PW-IDEAU100, Oviedo, Spain) were utilized in this study. These electrodes comprised 32 interdigitated fingers, each with a width and gap of 100 μm (see Figure 1). Bovine serum albumin (BSA), 25% glutaraldehyde in water, pure ethanol, and cysteamine were sourced from Sigma-Aldrich (Burlington, VT, USA). Phosphate-buffered saline (PBS) was obtained from Fisher Scientific (Waltham, MA, USA). IL-8 antibodies and antigens were procured from Abcam (Cambridge, UK). For the selectivity assessments, BSA, UL83 antigen (Miltenyi Biotec Ltd., Bisley, UK), and LDL antigen (Sino Biological, Beijing, China) were used.

### 2.2. Biofunctionalization of IL-8 Non-Faradaic Biosensor

The biosensing surface (interdigitated electrodes area denoted in Figure 1) was functionalized through a series of incubation and washing steps described in previous works [28,31,32]. Initially, the surface was incubated in a 1 mM ethanolic solution of cysteamine at 25 °C for 1 h in the dark. Following this, the electrode was rinsed with ethanol and dried using nitrogen. Subsequently, 50 µL of 2.5% glutaraldehyde in PBS was applied to the surface and incubated for 1 h, after which it was rinsed with deionized (DI) water and dried with nitrogen. For IL-8 antibody immobilization, 50 µL of a 1 µg/mL IL-8 antibodies solution in DI water was introduced and incubated for 1 h, followed by rinsing with DI water. Finally, surface blocking was performed by incubating the electrode with 50 µL of 5% bovine serum albumin (BSA) in PBS for 30 min. The biosensing surface was then washed with DI water and dried with nitrogen, preparing it for IL-8 antigen detection. The immobilization of these layers was confirmed in a previous work by different characterization methods [28,31,32]. Figure 1 illustrates the IL-8 antibody immobilization strategy employed in this work.

### 2.3. Non-Faradaic Detection of IL-8

Non-Faradaic electrochemical impedance spectroscopy (EIS) analysis was performed using a GAMRY Interface 1000™ potentiostat (GAMRY, Warminster, PA, USA). Initially, baseline measurements were obtained by introducing 50 µL of PBS onto the biosensing surface, followed by an open-circuit potential (OCP) measurement. OCP measurements were conducted to ensure system equilibrium by setting the direct current potential (VDC) to zero relative to the OCP, thereby minimizing potential bias between the two electrodes. The OCP was recorded for 100 s. Non-Faradaic EIS measurements were conducted over a frequency range of 200 mHz to 100 kHz, with an applied sinusoidal perturbation of 10 mV. Each measurement was repeated three times until a stable baseline was established. The PBS droplet was then removed by rinsing with DI water and drying with nitrogen. Subsequently, the biosensing surface was exposed to a series of IL-8 antigen solutions with concentrations ranging from 1 ng/mL to 10,000 ng/mL. After each incubation step, the surface was washed with DI water, with an incubation time of 5 min per antigen concentration. Changes in capacitance, impedance magnitude (Z_mod_), real impedance (Z_real_), and imaginary impedance (Z_imag_) were normalized to the sensor’s baseline response. To evaluate biosensor stability and detection limits, non-Faradaic measurements were also performed following successive incubations with PBS (blank). The sensitivity and limit of detection (LoD) were determined for the capacitance, Z_mod_, Z_real_, and Z_imag_. The biosensor’s selectivity was assessed by testing against positive samples containing IL-8 antigen, BSA, UL83 antigen, and LDL antigen. Additionally, a negative control sample containing only BSA, UL83 antigen, and LDL antigen (excluding IL-8 antigen) was analyzed to further validate selectivity.

## 3. Results and Discussion

**Figure 2:**
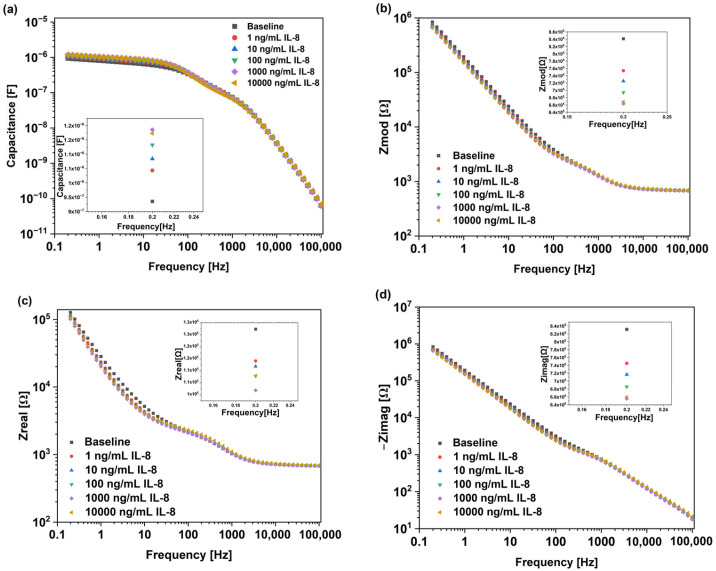
Bode plots of gold Au-IDE-based non-Faradaic impedimetric biosensors analyzed for IL-8 antigen detection, presenting data for (**a**) capacitance, (**b**) Z_mod_, (**c**) Z_real_, and (**d**) Z_imag_.

Figure 2a–d show the Bode plots of Au-IDE-based biosensor when tested against IL-8 antigens, in the frequency range of 200 mHz to 100 kHz. There were various but clear concentration-dependent responses to the IL-8 antigens across capacitance, Z_mod_, Z_real_, and Z_imag_. There was a continuous increase in capacitance with increasing the IL-8 antigen concentrations. The inset in Figure 2a demonstrates the measured capacitance of the sensor for the baseline and Il-8 concentration ranging from 1 ng/mL to 10,000 ng/mL at 0.2 Hz. A value of 0.2 Hz was chosen since maximum changes in impedimetric parameters were observed at this frequency. The baseline capacitance was measured to be around 9.6 × 10^−7^ F, which continuously increased with the increase in IL-8 concertation to 1.2 × 10^−6^ F at 10,000 ng/mL. The capacitance behavior here suggests an increase in charge accumulation at the interface, mostly because of the IL-8 binding to the sensor’s surface [33,34].

Figure 2b shows the steady decrease in Z_mod_ with increasing IL-8 antigen concentrations. At the lowest frequency (i.e., 0.2 Hz), the baseline value dropped from approximately 8.5 × 10^5^ Ω to around 6.9 × 10^5^ Ω at 10,000 ng/mL IL-8. The real impedance in Figure 2c shows, relative to the Z_mod_ and Z_imag_, smaller changes with a similar decreasing trend. The slight decreases in Z_real_ indicated that the impedance loss in the biosensor was low, which suggested the absence of Faradaic charge transfer at the interface. These findings suggested the absence of charge transfer reactions across the electrode interface, where the contribution of real impedance is often minimal [35].

To further assess the non-Faradaic behavior of the biosensor, Z_imag_ was analyzed as shown in Figure 2d. Z_imag_ followed a similar decreasing trend with larger changes in impedance compared to Z_real_. At 0.2 Hz, we could observe that the value of Z_imag_ for the baseline was approximately 8.4 × 10^5^, decreasing with the increase in IL-8 antigen concentration to 6.6 × 10^5^ at 10,000 ng/mL. The decrease in Z_imag_ was mainly due to the IL-8 antigen’s attachment to the surface, which impacted the system interaction with storing and transferring electrical charge. Hence, the observed impedance decreased. Z_imag_ is directly related to a sensor’s capacitive nature. These findings align with observations on non-Faradaic systems, where the capacitive response dominates the sensor behavior rather than the electron transfer reactions [28].

**Figure 3:**
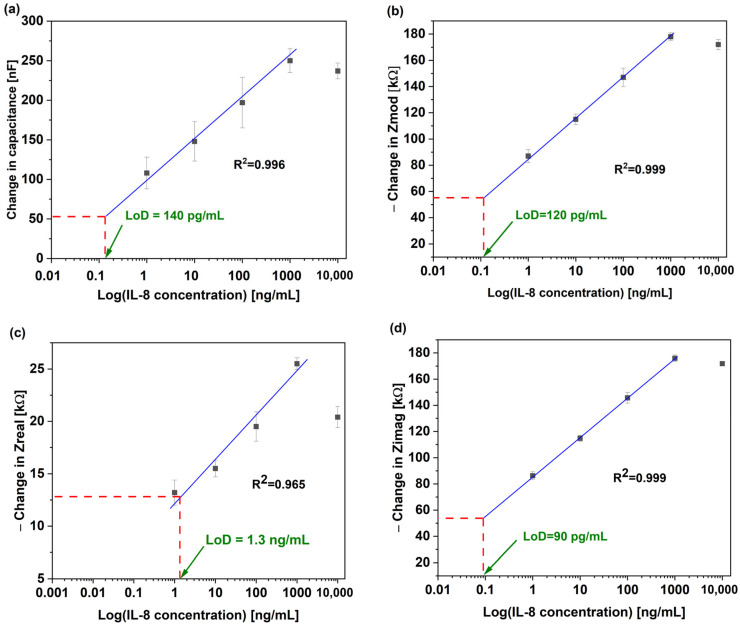
Au-IDE non-Faradaic biosensors’ calibration curves for (**a**) capacitance, (**b**) Z_mod_, (**c**) Z_real_, and (**d**) Z_imag_.

To assess the non-Faradaic sensor’s performance, calibration curves for the capacitance, Z_mod_, Z_real_, and Z_imag_ were plotted, as seen in Figure 3a–d. The data presented cover the linear fit of the data from Figure 2a–d, with an IL-8 concentration ranging from 1 ng/mL to 10,000 ng/mL. The data presented are based on measurements collected at 0.2 Hz. To explore the most sensitive impedimetric parameter of the biosensor, the limit of detection (LoD) for each parameter (capacitance, Z_mod_, Z_real_, and Z_imag_) was calculated using Equation (1), similar to previous studies [28,36,37]:

(1) $$X_{LoD}=f^{-1}(\underline{y}blank+3\sigma )$$

where $f^{-1}$ is the inverse function of the linear fit in Figure 3a–d, $\underline{y}blank$ is the mean readout of the control samples, and σ is the average standard deviation of the blank samples. The LoD of each parameter is found at the intersection between $\underline{y}baseline+3\sigma$ and the linear fitting line.

Z_imag_ showed the lowest LoD at 90 pg/mL, followed by Z_mod_ at 120 pg/mL, and capacitance at 140 pg/mL. On the other hand, Z_real_ showed the highest LoD at 1.3 ng/mL. The coefficient of determination (R^2^) for the relationship in these figures ranged between 0.965 and 0.999, which revealed the high reliability of the test.

**Figure 4:**
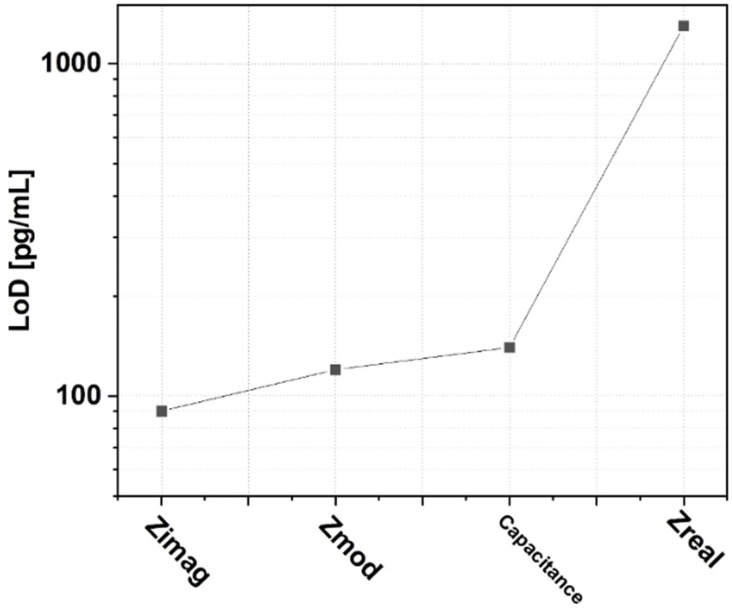
Obtained LoD value for each impedimetric parameter.

This study intended to observe the LoD of each parameter to explore the biosensors’ non-Faradaic behavior, with the lowest LoD observed being that of Z_imag_. Figure 4 clearly demonstrates the differences in Z_imag_ and Z_real_ in terms of the LoD (90 pg/mL and 1.3 ng/mL, respectively). This clearly indicates the non-Faradaic nature of the biosensor. The sensitivity of the biosensor is determined from the slope of the calibration curve from Figure 3a–d for each impedimetric parameter [28].

Table 1 shows the sensitivity of the biosensor for each measured parameter. Interestingly, Z_imag_ exhibited the highest sensitivity at 13.1 kΩ/log (ng/mL) among all measured parameters, which makes it the most effective for detecting IL-8 antigens at low concentrations. On the other hand, Z_real_ was less effective than the rest in detecting the IL-8 antigen, with the lowest sensitivity and highest LoD. A previous report indicated that gold biosensors are a great candidate for IL-8 detection with an LoD comparable to the detection norms [38]. Here, we confirm that the dominant behavior in gold biosensors is through the non-Faradaic interface. Impedance-based biosensors offer several advantages compared to conventional techniques such as rapid and label-free detection. Additionally, the lack of redox reactions in non-Faradaic sensors reduces electrode degradation, offering long-term stability [29]. Table 2 compares the reported sensor with others in the literature. While the other listed technologies are well established even commercially, further improvement is required for our biosensor to compete with their LoD. Nevertheless, the LoD for the reported sensor here is significantly below the clinical threshold of 600 pg/mL.

**Figure 5:**
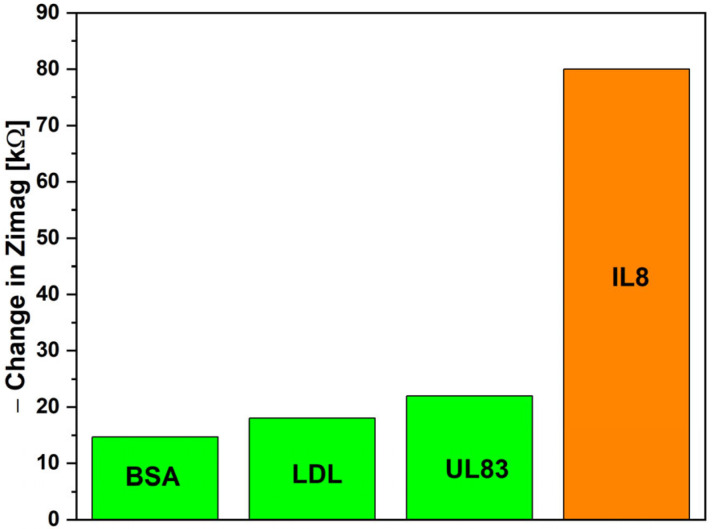
Selectivity performance of the biosensor.

Figure 5 presents the selectivity study conducted in this work. The biosensor was tested with separate samples, each containing 1 ng/mL of a specific antigen: BSA, LDL-antigen, UL83, or IL-8. Zimag was analyzed due to its superior biosensing performance. The observed changes in Zimag were 14, 17, 22, and 79 kΩ for BSA, LDL-antigen, UL83, and IL-8, respectively. These results highlight the biosensor’s exceptional selectivity for IL-8 antigens.

## 4. Conclusions

Overexpression and elevated levels of IL-8, a pro-inflammatory chemokine, are associated with a wide range of diseases. Because its concentration is routinely monitored for numerous medical disorders either for the purpose of rapid diagnosis or for prognosis [44], there is a crucial need for a non-invasive, rapid, and sensitive detection method.

A non-Faradaic Au-IDE-based biosensor for IL-8 detection was developed, evaluating multiple impedimetric parameters: capacitance, Z_mod_, Z_real_, and Z_imag_. The results appear auspicious, specifically the finding that the Z_imag_ parameter offers the lowest LoD and the highest sensitivity. Our findings highlight the need for a comprehensive analysis of multiple impedimetric parameters rather than relying solely on capacitance. While non-Faradaic biosensors are fundamentally based on capacitive sensing, alternative parameters may offer superior sensitivity and detection limits. This study provides valuable insights into the optimization of non-Faradaic impedimetric biosensors for biomarker detection, offering a simple and scalable approach for the direct and high-throughput screening of disability and inflammation-related biomarkers.

These findings are significant for point-of-care diagnosis, where extreme sensitivity and a low LoD, together with rapid results, are necessary to permit early diagnostics. Further validation is needed using real patient samples to confirm the results and a comparison with ELISA to validate the biosensor’s performance. Nevertheless, the findings highlight a promising step towards a non-invasive, rapid, and sensitive detection method for IL-8.

## Figures and Tables

**Table 1:** LoD and sensitivity obtained for each impedimetric parameter.

	LoD	Sensitivity
Zimag	90 pg/mL	13.1 kΩ/log (ng/mL)
Zmod	120 pg/mL	13.2 kΩ/log (ng/mL)
Capacitance	140 pg/mL	20 nF/log (ng/mL)
Zreal	1.3 ng/mL	1.8 kΩ/log (ng/mL)

**Table 2:** Limit of detection of IL-8 using different techniques.

Technique	LoD [pg/mL]	Ref.
Au-IDE non-Faradaic biosensor	90	This work
Enzyme-linked immunosorbent assay	1.1	[39]
Lumit^®^ IL-8 immunoassay	1	[40]
Single-molecule array (Simoa) assay	0.056	[41]
HTRF human IL-8 kit	6	[42]
Chemiluminescence	2	[43]

## Data Availability

The original contributions presented in this study are included in the article. Further inquiries can be directed to the corresponding author.
